# Discrepancy in diagnoses of diabetes and prediabetes using fasting plasma glucose and glycosylated hemoglobin and the underdiagnosis by ICD-10 coding: data from a tertiary hospital in Thailand

**DOI:** 10.3389/fpubh.2023.1322480

**Published:** 2023-12-13

**Authors:** Napalai Poorirerngpoom, Poranee Ganokroj, Arnond Vorayingyong, Thanapoom Rattananupong, Jennifer Pusavat, Thanan Supasiri

**Affiliations:** ^1^Department of Preventive and Social Medicine, Faculty of Medicine, Chulalongkorn University, Bangkok, Thailand; ^2^Department of Laboratory Medicine, Faculty of Medicine, Chulalongkorn University, Bangkok, Thailand; ^3^Michigan State University College of Osteopathic Medicine, East Lansing, MI, United States; ^4^Life Center, Samitivej Sukhumvit Hospital, Bangkok, Thailand

**Keywords:** diabetes mellitus, prediabetes, HbA1c, fasting plasma glucose, diagnosis, ICD-10

## Abstract

**Background:**

Early detection of prediabetes and diabetes better prevents long-term health complications. FPG and HbA1c levels are some common laboratory tests utilized as tools to diagnose diabetes and prediabetes, but the agreement rate between these two diagnostic tests varies, which could lead to underdiagnosis and thus undertreatment. This study aimed to analyze the agreement rate between FPG and HbA1c, as well as the physicians’ accuracy of using these results to make a prediabetes or diabetes diagnosis through ICD-10 coding at a tertiary care hospital in Bangkok, Thailand.

**Methods:**

A cross-sectional descriptive study was conducted using secondary data collected in a tertiary hospital’s check-up clinic from August 16, 2019 to June 30, 2022 to study the prevalence and diagnosis of diabetes and prediabetes, determined through FPG and HbA1c laboratory results. We analyzed the two laboratory tests’ diagnosis agreement rate and the physicians’ accuracy of diagnosing diabetes and prediabetes in ICD-10 coding using the FPG and HbA1c results.

**Results:**

Among 8,024 asymptomatic participants, the period prevalence diagnosed through laboratory results was 5.8% for diabetes and 19.8% for prediabetes. Diabetes and prediabetes prevalence based on laboratory data differs from that of ICD-10 coding data. Specifically, 79.6% of diabetes patients and 32.3% of prediabetes patients were coded using the ICD-10 coding system. 4,094 individuals had both FPG and HbA1c data. The agreement rate for diagnosing diabetes and prediabetes between the two laboratory results is 89.5%, with Kappa statistics of 0.58. Using only one of the two laboratory results would have missed a substantial number of patients.

**Conclusion:**

Our findings highlight screening test discrepancies and underdiagnosis issues that impede diagnostic accuracy enhancement and refined patient management strategies. Early diagnoses of prediabetes and diabetes, especially before symptoms arise, could increase health consciousness in individuals, thereby enabling the implementation of lifestyle modifications and prevention of serious health complications. We emphasize the importance of diagnosing these conditions using both FPG and HbA1c, along with subsequent accurate ICD-10 coding. Even though some hospitals lack certified HbA1c testing, we suggest enhancing the availability of HbA1c testing, which could benefit many people in Thailand.

**Clinical trial registration**:

https://www.thaiclinicaltrials.org, identifier [TCTR20230824003].

## 1 Introduction

Diabetes mellitus (DM) is a prevalent non-communicable disease that represents a substantial global public health concern. It is associated with numerous complications, such as cardiovascular disease, neuropathy, retinopathy, and nephropathy, which contribute to increased morbidity and mortality rates. Early detection and diagnosis of prediabetes and diabetes are crucial for effective management and facilitating lifestyle modifications aimed at preventing or delaying the onset of these complications.

The American Diabetes Association (ADA) recommends several diagnostic tests for prediabetes and diabetes, including fasting plasma glucose (FPG), 2-h plasma glucose (2-h PG) during a 75-gram oral glucose tolerance test (OGTT), glycosylated hemoglobin (HbA1c), or the presence of classic symptoms indicative of hyperglycemia or a hyperglycemic crisis with random plasma glucose as a supporting measure (1). However, the agreement rates between these diagnostic tests vary (2). For example, Karnchanasorn et al. conducted a study to compare the effectiveness of HbA1c with FPG and 2-h PG in diagnosing diabetes in a previously undiagnosed diabetic cohort and found that the sensitivity of HbA1c ≥ 6.5% was only 43.3% compared to the FPG criterion and 28.1% compared to the 2hPG criterion (3). On the contrary, Lim et al. compared HbA1c and FPG as diabetes screening modalities in Singapore residents of Chinese, Malay and Indian race to detect diabetes mellitus and showed that HbA1c is an effective alternative to FPG and combining HbA1c with FPG measurements can further improve diabetes detection. (4)

Moreover, the accurate coding of DM according to the International Classification of Diseases, Tenth Revision (ICD-10) is essential for recording, reporting, and monitoring diseases (5, 6). Inadequate or incorrect coding can lead to underdiagnosis or misclassification, resulting in erroneous national prevalence calculations and an inaccurate burden of disease implications. Despite efforts to improve coding practices, discordance may still occur between recorded ICD-10 codes and the actual laboratory diagnosis of DM in clinical settings (7).

Generally, the HbA1c test that is recommended for use as DM diagnostic tool should be a certified method by National Glycohemoglobin Standardization Program (NGSP), which verifies the HbA1c results to the Diabetes Control and Complications Trial (DCCT) and United Kingdom Prospective Diabetes Study (UKPDS), as these cohort studies had established association between HbA1c levels and outcome risks in patients with DM. In Thailand, few laboratories lack certified HbA1c testing, but the diagnosis of prediabetes and diabetes still predominantly relies on only FPG.

In light of these diagnostic practices, the present study aims to investigate the variation in diagnosing prediabetes or impaired fasting glucose (IFG) and diabetes using FPG and HbA1c within a tertiary hospital setting in Thailand. By assessing the prevalence and magnitude of these discrepancies, this study intends to provide valuable insights into Type 2 diabetes mellitus (T2DM) diagnosis and coding practices. Moreover, the study will address the issue of underdiagnosis associated with ICD-10 coding and explore the potential discordance between the recorded ICD-10 codes and laboratory-confirmed T2DM diagnoses. These findings will contribute to improving accuracy and reliability in T2DM diagnoses, enabling better patient management and prevention of diabetic complications.

## 2 Materials and methods

A cross-sectional descriptive study was conducted using secondary data collected in a tertiary hospital’s check-up clinic to study the prevalence and diagnosis of diabetes and prediabetes. The study was conducted through two comparable data sources: ICD-10 coding data recorded by physicians at the health check-up clinic from Information Technology Department and laboratory results from the Department of Laboratory Medicine. Data were drawn by selected all individuals that visited the clinic from August 16, 2019 to June 30, 2022, for a period of 2 years, 10 months, and 16 days.

At this health check-up clinic, patients are recommended and offered two different screening programs, including laboratory checklists, based on their age. The first program is for people who are age 35 and older. This program includes a comprehensive laboratory checklist that consists of a complete blood count (CBC), fasting plasma glucose (FPG) level, aspartate aminotransferase (AST), alanine aminotransferase (ALT), alkaline phosphatase (ALP), blood urea nitrogen (BUN), creatinine, lipid profile (total cholesterol, triglyceride, HDL-cholesterol, and calculated LDL-cholesterol), uric acid, and urinalysis. The second program is for those under the age of 35, and the laboratory items for this age group include a CBC and urinalysis. However, individuals can choose to include additional tests such as glycosylated hemoglobin (HbA1c) and other items. All laboratory tests mentioned above were performed at a certified standardized laboratory at the Department of Laboratory Medicine, King Chulalongkorn Memorial Hospital, which used the certified method for HbA1c testing and also is a certified laboratory accredited by NGSP.

This study was approved by the Institutional Review Board (IRB), Faculty of Medicine, Chulalongkorn University (COA No. 0573/2565), and it was approved for registration at Thai Clinical Trials Registry (TCTR identification number: TCTR20230824003).

### 2.1 Data collection

Data was collected from all individuals who visited our check-up clinic and underwent an annual health check-up. This included a thorough medical history, physical examination, laboratory testing, and ICD-10 coding. For this study, we obtained two sets of data, which were provided to us in Microsoft Excel (.xlsm) formats. The two data sets used in this study were as follows:

1. ICD-10 coding dataset from the Information Technology Department, which included the clinic code, clinic name, date of examination, ID, sex, age (in years, determined by the date of examination), ICD10 codes, and diagnosis names.
2. Laboratory dataset from the Department of Laboratory Medicine, which contained the lab number, order date, collection date, ID, sex, birth date, age, source code, source description, and FPG and HbA1c measurements.

Data preprocessing: After acquiring both datasets, they were merged using each individual’s ID with the STATA version 17.0 program. Incomplete data was removed, and if an individual received health check-ups more than once during the study period, the most recent recorded data was selected for study participation.

### 2.2 Data analysis

The prevalence of prediabetes and diabetes were determined in this study using descriptive statistics. The agreement rate and discrepancy in diagnoses of diabetes and prediabetes between FPG and HbA1c were analyzed using descriptive and Kappa statistics. We also compared diagnoses of diabetes and prediabetes from doctors’ ICD-10 coding and laboratory results using descriptive statistics.

Individuals were evaluated as diabetic from disease coding if physicians coded ICD-10 codes beginning with E10-E14. Individuals were evaluated as diabetic from laboratory tests if their datasets included at least one of the following: FPG ≥ 126 mg/dL or HbA1c ≥ 6.5% (1).

Individuals were evaluated as prediabetic from disease coding if physicians coded ICD-10 codes beginning with R73. Individuals were evaluated as diabetic from laboratory tests if their datasets included at least one of the following: FPG range of 100–125 mg/dL or a HbA1c range of 5.7–6.4% (1).

## 3 Results

There were 8,024 individuals who came for an annual check-up from August 16, 2019 to June 30, 2022 at the check-up clinic. The mean age of the individuals was 52.3 ± 14.3 years, and 66.2% were female (Table 1).

**Table 1:** Demographic data of the individuals.

Characteristic	Number	%
All patients	(*n* = 8024)	
Mean age (year), (SD)	52.3 (14.3)	
Male gender	2,712	33.8
Patients who tested both FPG and HbA1c	(*n* = 4,094)	
Mean age (year), (SD)	53.4 (13.5)	
Male gender	1,441	35.2

Of the 8,024 individuals, using their FPG or HbA1c results, we found 466 (5.8%) and 1,586 (19.8%) individuals that met criteria for diabetes and prediabetes, respectively. However, the prevalence identified through ICD-10 coding data was 4.6% for diabetes and 6.4% for prediabetes. Of the patients that met criteria, 79.6% (371/466) of diabetic patients and 32.3% (512/1586) of prediabetic patients were diagnosed by the ICD-10 coding system (Table 2).

**Table 2:** Prevalence of diabetes and prediabetes.

	diagnosed from ICD-10 coding system	diagnosed by laboratory results
Diabetes	4.6% (371/8024)	5.8% (466/8024)
Prediabetes	6.4% (512/8024)	19.8% (1,586/8024)

Given that FPG is an optional test for individuals under the age of 35, and HbA1c is optional for all checkup patients, Table 3 presents the prevalence of diabetes and prediabetes across three data categories: (1) individuals with only FPG results, (2) individuals with only HbA1c results, and (3) individuals with both FPG and HbA1c results. The prevalence rates were found to be 3.6, 4.6, and 7.8% for diabetes and 12.7, 11.4, and 26.6% for prediabetes, respectively.

**Table 3:** Prevalence of diabetes and prediabetes from laboratory results.

	diagnosed from FPG only (No HbA1c tested)	diagnosed by HbA1c only (No FPG tested)	diagnosed by FPG and/or HbA1c (had both FPG and HbA1c results)
Diabetes	3.7% (143/3886)	4.6% (2/44)	7.8% (321/4094)
Prediabetes	12.7% (494/3886)	11.4% (5/44)	26.6% (1,087/4094)

There were 4,094 individuals with both FPG and HbA1c laboratory results. Out of these 4,094 individuals, there were 321 (7.8%) patients with diabetes and 1,087 (26.6%) patients with prediabetes. Table 4 details the distribution of FPG and HbA1c results into diagnosis categories. Using FPG alone for diagnosis, there would be 236 patients with diabetes (FPG ≥ 126 mg/dL) and 672 patients with prediabetes (FPG: 100–125 mg/dL). Using HbA1c alone for diagnosis, there would be 280 patients with diabetes (HbA1c ≥ 6.5%) and 897 patients with prediabetes (HbA1c: 5.7–6.4%). Moreover, 574 of these patients were undiagnosed using FPG alone.

**Table 4:** Number of individuals (percent) with FPG and HbA1c.

		Fasting plasma glucose			Total
		< 100 mg/dL	100–125 mg/dL	≥ 126 mg/dL	
HbA1c	< 5.7%	2,686 (65.61)	227 (5.54)	4 (0.10)	2917
	5.7–6.4%	489 (11.94)	371 (9.06)	37 (0.90)	897
	≥ 6.5%	12 (0.29)	73 (1.78)	195 (4.76)	280
	Total	3,187	672	236	4094

The agreement rate of diagnosing diabetes and prediabetes between FPG and HbA1c is 89.5%, with Kappa statistics of 0.58, value of *p* 0.01.

## 4 Discussion

From the demographic data (Table 1) of our study’s participants, the majority of individuals (66%) in this health check-up clinic study were female. This finding differs from population data from the National Statistical Office website, which indicates that females constitute 53% of the population and housing accessed in Bangkok in 2021 (2,935,702/5,527,994). These results suggest that females may be more conscientious in seeking out health check-ups than males.

From Table 2, 20.4% (95/466) of diabetic patients and 67.7% (1,075/1586) of prediabetic patients were misdiagnosed by ICD-10 coding. Thus, if only using data from ICD-10 coding, a lot of diabetic patients and the majority of prediabetic patients would be undiagnosed and may not receive adequate education and treatment. The notable discordance between the coding data and laboratory data for prediabetes prevalence rates may suggest that clinicians do not prioritize the condition and as such do not assign a diagnosis code for it. Additionally, the data also showed that for prediabetic patients diagnosed by HbA1c alone, only 5.8% were coded by doctors’ ICD-10 coding. This represents an opportunity for the hospital to emphasize this diagnostic approach to healthcare practitioners and increase the coding rate for this condition.

The results from Table 3 reveal important insights regarding the prevalence of diabetes and prediabetes among individuals who underwent different choice of screening tests at the health check-up clinic. Notably, we observed varying prevalence rates for diabetes and prediabetes among the three data categories we analyzed, with the highest rates being evident in individuals who received both FPG and HbA1c tests. This underscores the potential benefit of combining these tests to achieve more accurate diabetes and prediabetes detection. Furthermore, our data from Table 4 revealed that there were only 4,094 out of 8,024 individuals with both FPG and HbA1c laboratory results. From these results, if using HbA1c alone for diagnosis, up to 12.8% (41/321) of diabetic patients and 20.9% (227/1087) of prediabetic patients would have been missed. In contrast, using FPG alone for diagnosis, we would miss up to 26.5% (85/321) of diabetic patients and up to 45.0% (489/1087) prediabetic patients, almost half of them.

Although this differs from other findings that HbA1c detects diabetes less often than FPG (8), we propose the integration of HbA1c testing into routine check-ups for the Thai population. Even with a certified lab, relying solely on FPG testing without incorporating HbA1c would lead to significant underdiagnosis of diabetes and prediabetes cases as previously discussed.

Considering the potential inclusion of HbA1c testing for diabetes screening during check-ups, the associated direct cost to identify an additional case of prediabetes or diabetes is approximated at 1069.9 Thai baht. This estimation is based on the fact that each HbA1c laboratory test at this tertiary hospital costs 150 Thai baht (the amount as set by The Comptroller General’s Department of Thailand), covering 4,094 cases tested to identify 574 additional cases of these conditions. The number needed to test is 7.1 cases (4,094/574). Given these insights, we recommend adding HbA1c testing to enhance the screening program’s effectiveness. This recommendation aligns with evidence indicating its cost-effectiveness over FPG, especially in regions where research discloses a significantly higher prevalence of cases identified by HbA1c compared to FPG (9). Our study likewise confirms a higher prevalence of cases identified by HbA1c as opposed to FPG.

We recommend the inclusion of HbA1c testing as an additional measure to identify a higher number of cases of prediabetes and diabetes. This implementation would facilitate timely interventions and subsequently delay disease progression. The significance of early detection lies in its ability to allow for tailored lifestyle interventions, thereby mitigating the risk of diabetes. Notably, established guidelines, exemplified by the American Diabetes Association (ADA), underscore the importance of diagnosing prediabetes (10), with HbA1c standing out as a reliable diagnostic tool due to its capacity to reflect extended glucose control (11). By integrating HbA1c testing into routine health check-ups, the ability to detect prediabetes is significantly enhanced, creating an avenue for prompt interventions such as dietary modifications and exercise, both of which have been empirically proven by the U.S. National Diabetes Prevention Program (DPP) to markedly reduce the risk of diabetes during the prediabetes stage and consequently lower the incidence of diabetes. This evidence-based lifestyle change program developed by the Centers for Disease Control and Prevention (CDC) is tailored to individuals with prediabetes or those at risk of developing type 2 diabetes (12). Operating within diverse community settings like healthcare organizations, workplaces (13), and primary care centers (14), the DPP serves as a program guideline for diabetes prevention. By focusing on proactive interventions and lifestyle modifications, this program plays a vital role in preventing or delaying the onset of T2DM, ultimately contributing to the broader endeavor of public health advocacy (15).

We maintain using both FPG and HbA1c in diabetes and prediabetes screening, since HbA1c results are influenced by various factors, including conditions affecting red cell turnover and hemoglobin levels, such as hemolytic anemias, glucose-6-phosphate dehydrogenase deficiency, blood transfusion, erythropoiesis-stimulating drug use, end-stage kidney disease, and pregnancy (16). Notably, hemolytic anemia or blood loss is associated with lower HbA1c levels, while iron deficiency anemia is linked to higher levels (17). Although specific information about these conditions in our patient population is lacking, our data on hemoglobin (Hb) levels reveal significant differences among individuals with normal HbA1c, elevated HbA1c, and diabetes-range HbA1c, exhibiting an increasing trend (Table 5). Further research is needed to explore the precise impact of these factors on HbA1c measurements, contributing valuable insights to enhance the accuracy and interpretation of HbA1c as a diagnostic tool in clinical practice.

**Table 5:** Association between HbA1C and Hb level.

		HbA1c (%) [mean (SD)]	Hb (g/dl) [mean (SD)]
HbA1c	normal < 5.7%	5.2 (0.3)	13.4 (1.5)
	prediabetes range 5.7-6.4%	5.9 (0.2)	13.6 (1.3)
	diabetes range ≥ 6.5%	8.3 (2.1)	13.9 (1.8)

The study’s limitations included possible overestimated prevalence rates since we considered at least a single laboratory test in order to classify a diagnosis of diabetes or prediabetes. According to DM diagnosis guidelines, a more accurate diagnosis would only apply to individuals with two abnormal diabetes screening test results (2). Even so, our findings highlighted screening test discrepancies between the two tests that potentially impede diagnostic accuracy enhancement and refined patient management strategies. In particular, we investigated the prevalence of diabetes and prediabetes among asymptomatic individuals who underwent health check-ups. Thus, the prevalence rate of 5.8 and 19.8% for diabetes and prediabetes laboratory results mainly reflected the study population in those who had self-voluntary came for health check-up with the conditions that FPG is optional for individuals under 35 and HbA1c is optional for all checkup patients according to the reimbursement criteria of The Comptroller General’s Department of Thailand. Therefore, the prevalence rate might differ from the general Thai population, which has the prevalence of diabetes ranging from 9.6–9.7% and prediabetes ranging from 7.0–10.6% (18–21).

In conclusion, since early diagnosis of prediabetes and diabetes would increase health-consciousness in individuals to implementing lifestyle modification and prevent further complications, we emphasize making diagnoses using both FPG and HbA1c and accurately coding ICD-10. As some hospital laboratories do not offer certified HbA1c, we also suggest increasing the availability of HbA1c testing, which would be beneficial to most people. Moreover, a comprehensive ICD-10 coding is essential for improving public health policies. It can be used to track the prevalence of diseases and risk factors, identify disparities in healthcare access and utilization, and evaluate the effectiveness of public health programs. However, our study found that ICD-10 coding inaccuracy is more common than previously thought, especially for conditions that may not require medication prescriptions, such as prediabetes, which is often overlooked. To address this problem, we recommend regular communication with doctors to emphasize the importance of accurate coding, providing coding support tools such as coding resources and guidelines specifically related to prediabetes, implementing coding audits and providing feedback to individual providers and healthcare organizations, and targeted education or training.

### Scope statement

In this study, we address key aspects of diagnosing Type 2 diabetes mellitus (T2DM) with a primary focus on its early identification and the consequential impact on clinical practice. Given the well-established recognition of T2DM as a formidable public health challenge, our study uncovers novel insights derived from an investigation conducted within a Thai tertiary hospital. We illuminate significant discrepancies in diagnosing prediabetes and diabetes, employing fasting plasma glucose (FPG) and glycosylated hemoglobin (HbA1c) measurements. Moreover, we address the pressing issue of underdiagnosis as evident in ICD-10 coding practices, which could inadvertently lead to an underestimated disease burden within the country.

The discrepancy between recorded ICD-10 codes and laboratory diagnoses emphasizes the importance of adopting meticulous diagnostic and coding practices to ensure accurate representation. Additionally, we highlight the risk of underdiagnosis with FPG alone. Therefore, we recommend integrating HbA1c screening, especially in Thailand where a substantial proportion of prediabetic patients exhibit elevated HbA1c levels despite having normal FPG levels. This aligns with the goal of preventing diabetes and its complications through early identification and targeted lifestyle intervention strategies.

This submission contributes profound insights into reshaping diabetes and prediabetes diagnostic approaches, clinical decisions, and future healthcare strategies in the Thai context.

## Data availability statement

The data analyzed in this study is subject to the following licenses/restrictions: the data analyzed in this study belongs to King Chulalongkorn Memorial Hospital (KCMH). Access to the data must be approved by KCMH. Requests to access these datasets should be directed to TS, thanan.s@chula.ac.th.

## Ethics statement

The studies involving humans were approved by The Institutional Review Board (IRB), Faculty of Medicine, Chulalongkorn University. The studies were conducted in accordance with the local legislation and institutional requirements. Written informed consent for participation was not required from the participants or the participants' legal guardians/next of kin in accordance with the national legislation and institutional requirements.

## Author contributions

NP: Conceptualization, Data curation, Formal analysis, Methodology, Project administration, Validation, Writing – review & editing. PG: Conceptualization, Methodology, Validation, Writing – review & editing. AV: Conceptualization, Writing – review & editing. TR: Conceptualization, Formal Analysis, Methodology, Writing – review & editing. JP: Writing – review & editing. TS: Conceptualization, Formal analysis, Funding acquisition, Methodology, Project administration, Supervision, Validation, Writing – review & editing.
